# Elevated expression of BAFF receptor, BR3, on monocytes correlates with B cell activation and clinical features of patients with primary Sjögren’s syndrome

**DOI:** 10.1186/s13075-020-02249-1

**Published:** 2020-06-23

**Authors:** Keiko Yoshimoto, Katsuya Suzuki, Eriko Takei, Yumi Ikeda, Tsutomu Takeuchi

**Affiliations:** 1 Division of Rheumatology, Department of Internal Medicine, Keio University School of Medicine, 35 Shinanomachi, Shinjuku, Tokyo, 160-8582 Japan; 2 Keio University Hospital Clinical and Translational Research Center, 35 Shinanomachi, Shinjuku, Tokyo, 160-8582 Japan

**Keywords:** Primary Sjögren’s syndrome, BAFF, BR3, IL-6, IgG, Monocytes, B cell

## Abstract

**Background:**

We reported that the production of BAFF (B cell-activating factor) and IL-6, both of which are involved in survival and differentiation of B cells, is dysregulated in monocytes of patients with primary Sjögren’s syndrome (pSS). In this study, we investigate the relationship between possible aberrations of pSS monocytes and clinical features of pSS patients and the contribution of monocytes to B cell activation, a mechanism involved in the pathogenesis of pSS.

**Methods:**

Expression of BAFF-receptor (BR3) on peripheral monocytes from patients with pSS (*n* = 67) and healthy controls (HC: *n* = 37) was analyzed by FACS. Peripheral monocytes were stimulated with BAFF, and IL-6 production by the cells was measured by ELISA. Peripheral B cells were cultured with BAFF-stimulated monocytes in the presence or absence of anti-IL-6 receptor antibody, and IgG production by the cells was measured by ELISA. Patients’ serological data were collected from their clinical records. Patients’ disease activity was quantified based on their EULAR Sjögren’s syndrome disease activity index (ESSDAI) scores.

**Results:**

The proportion of peripheral BR3-positive monocytes (BR3^+^/CD14^+^) was significantly increased in pSS patients compared to HC. Moreover, IL-6 production by BAFF-stimulated monocytes was remarkably higher than HC and was significantly correlated with BR3^+^/CD14^+^ ratios of patients. In addition, BR3 expression on pSS monocytes was elevated in anti-Ro/SSA and/or anti-La/SSB positive compared to negative patients. Remarkably, BR3 expression on peripheral monocytes was positively and significantly correlated with patients’ serum IgG and IgM levels and ESSDAI scores. Moreover, the amount of IgG produced by B cells was markedly higher in pSS patients compared to HC when the cells were co-cultured with BAFF-stimulated autologous monocytes in vitro. Notably, addition of anti-IL-6 receptor antibody into the co-culture system led to inhibition of IgG production by B cells.

**Conclusions:**

Our data suggest that elevated BR3 expression in monocytes is associated with clinical features in pSS patients and that enhanced production of IL-6 by BAFF-stimulated monocytes plays a part in the overproduction of IgG by B cells in pSS. These results suggest that BAFF signaling pathways through BR3 in monocytes are possible therapeutic targets for pSS.

## Introduction

Primary Sjögren’s syndrome (pSS) is an idiopathic autoimmune disease with major clinical manifestations comprising xerostomia (dry mouth) and keratoconjunctive sicca (dry eyes) and is often accompanied with hypergammaglobulinemia (HγG). Several lines of evidence suggest that focal lymphocytic infiltration of the exocrine glands is responsible for lesion formation, IgG production, and subsequent dysfunction of the glands [1].

Many studies have shown that abnormal B cell functions, such as those that lead to conditions like appearance of rheumatoid factor, HγG, and anti-Ro/SSA and anti-La/SSB antibodies, are a hallmark of pSS [2]. In addition, the proportion of peripheral B cell subsets, such as CD27^+^IgM^+^IgD^+^ and CD27^+^IgM^−^IgD^−^ memory B cells, are reduced in pSS compared to healthy controls (HC) and patients with rheumatoid arthritis and systemic lupus erythematosus [3, 4], suggesting that these memory B cell subsets may be recruited to glandular tissues, where they differentiate into plasmablasts and plasma cells and produce IgG [5]. We previously reported that the proportion of peripheral CD38^high^IgD^+^ B cells, pre-germinal center B cells, was increased in pSS patients and this elevation was strongly correlated with patients’ serum IgG level and ESSDAI score [6]. Therefore, elucidation of the mechanisms of B cell activation may lead to the discovery of promising therapeutic targets for pSS.

BAFF (B cell-activating factor or tumor necrosis factor ligand superfamily, member 13b) is a cytokine that promotes proliferation, differentiation, and survival of B cells [7]. BAFF is a type-II membrane-bound protein, with a C-terminal fragment (soluble BAFF: or sBAFF) that can be released from the cells [7] and bind to its receptors (i.e., TACI (transmembrane activator and calcium-modulator and cyclophilin ligand (CAML) interactor), BCMA (B-cell maturation antigen) and BR3) [8–10]. BAFF is mainly produced by monocytes, dendritic cells [11–13], and T cells [7, 14]. We have been investigating the possible involvement of BAFF in the pathogenesis of pSS because BAFF plays a pivotal role in B cell activation [7, 11, 13, 15] and is involved in the pathogenesis of diseases associated with antibody formation, such as pSS. For example, several groups have reported that BAFF levels are elevated in the serum of pSS patients [16–18] and that this elevation is correlated with the patients’ serum IgG and autoantibody levels, such as anti-Ro/SSA and anti-La/SSB antibodies [19, 20]. Moreover, BAFF is strongly expressed in lymphocytes that infiltrate the salivary glands [21, 22].

The BAFF receptor, BR3, is also known to be deeply involved in processes related to B cell activation, such as the proliferation and differentiation of B cells [23, 24]. Therefore, BAFF and BR3 are promising targets for the treatment of the disease, and several clinical trials of drugs targeting BAFF (belimumab, an anti-BAFF mAb) and BR3 (VAY736, an anti-BR3 mAb) for pSS have been conducted for pSS [25–27]. In our previous study, we reported that BR3 expression in peripheral monocytes was elevated in pSS patients compared to HC and that pSS monocytes showed robust increases in IL-6 production following BAFF stimulation [28]. In addition, we revealed that the expression levels of transcription factors related to the expression of IL-6 were abnormally elevated in pSS monocytes [29]. Moreover, other studies have reported that systemic and/or local concentrations of IL-6 are also significantly elevated in pSS patients compared to HC [30, 31]. These findings may provide clues for elucidation of the mechanism underlying the pathogenesis of pSS, given that IL-6 promotes B cell differentiation [29], which is responsible for the production of autoantibodies.

However, the relationship between the abnormalities of peripheral cells and the clinical features of pSS is not fully understood. In the present study, we focused on the aberrations of pSS monocytes and investigated the possible involvement of monocytes in the pathogenesis of pSS.

## Materials and methods

### Patients and controls

Venous blood samples were collected from pSS patients (*n* = 67; all female, age 34–87 years (mean 61.1)) and HC (*n* = 37; all females, age 24–57 years (mean 41.6)). The pSS patients enrolled in this study fulfilled at least one of the following criteria: 2002 American-European criteria for SS (AECG) [32], 2012 American College of Rheumatology classification criteria for SS (ACR) [33], or the revised Japanese Ministry of Health criteria for the diagnosis of SS [34]. At the time of blood collection, five patients were receiving prednisolone at a daily dose of 4 to 10 mg, while the remaining patients were free of medication. Informed consent was obtained from each participant enrolled in this study before blood collection. This study was approved by the ethics committees of Keio University School of Medicine (#20100080, #20140335).

### FACS analysis

FACS analysis of the cells in whole blood was performed according to methods recommended by the manufacturer of the antibodies (BD Biosciences). The expression level of BR3 in peripheral monocytes was analyzed by FACS according to previously described method [28] Briefly, to determine the proportion of BR3^+^ cells among CD14^+^ monocytes, 50 μl of whole blood sample was incubated with PE-Cy7-labeled anti-human CD14 antibody and PE-labeled anti-human BR3 antibody or PE-labeled anti-human TACI antibody for 30 min at room temperature. The cells were subsequently lysed and fixed by Lyse/Fix Buffer (BD Biosciences, San Jose, CA, USA) and examined using MACS Quant Analyzer (MACS Quant Analyzer®, Miltenyi Biotec). The proportion of BR3^+^ cells among CD14^+^ monocytes was defined as shown in Supplementary Fig. 1.

### Cell culture

Peripheral monocytes and B cells were isolated from whole blood samples by using anti-CD19 antibody-coated microbeads and anti-CD14 antibody-coated microbeads, respectively (Miltenyi Biotec, Bergisch Gladbach, Germany) with an autoMACS® Separator (Miltenyi Biotec). FACS analyses showed that the purity was > 90% for both cell types. The effect of sBAFF on IL-6 production by monocytes was investigated as described previously [28]. Briefly, monocytes (2.5 × 10^5^/ml) prepared from pSS patients and HC were cultured in vitro for 96 h in the presence of 2.0 μg/ml of recombinant human sBAFF. Subsequently, the concentration of IL-6 in the culture supernatants was measured by sandwich ELISA. To examine the effects of monocytes on IgG production, B cells (7.5 × 10^4^/ml) from an individual pSS patient were mixed with monocytes (1.5 × 10^5^/ml) prepared from the same patient. The ratio of B cells and monocytes in the co-culture was determined according to the actual ratio of these cells in the peripheral blood of pSS patients, which was estimated using FACS (data not shown). The cells were cultured in a 24-well plate with a traswell insert in the absence or presence of 2.0 μg/ml of sBAFF for 4 days.

### Antibodies and recombinant protein

Recombinant human soluble BAFF was purchased from Peprotech (Rocky Hill, USA). The anti-human IL-6 receptor mAb for cell culture, anti-human IgG antibody for ELISA, and Pacific Blue (PB)-labeled anti-CD14 mAb, phycoerythrin (PE)-labeled anti-TACI mAb, and PE-labeled anti-BR3 antibody for FACS were purchased from BD Biosciences (San Jose, CA, USA).

### ELISA

The amount of IL-6 by monocytes and IgG produced by B cells co-cultured with autologous monocytes in the presence or absence of sBAFF in vitro was measured using ELISA according to methods recommended by the manufacturer of the antibodies (BD Bioscience).

### Statistical analysis

Differences between the groups were statistically analyzed using the two-tailed Student’s *t* test for single comparison, unless otherwise noted. Pearson’s correlation analysis was employed to evaluate the linear relationship between two continuous variables. A *p* value of less than 0.05 was used to indicate a statistically significant difference.

## Results

### Laboratory parameter abnormalities of pSS patients

We investigated the laboratory parameters of the pSS patients enrolled in this study (Table 1). The average IgG serum level was 1653 ± 587 mg/dl, and 32.8% of patients had serum IgG levels above the reference range of normal Japanese individuals (870–1700 mg/dl) [35] (Table 1). Although pSS patients aged 65 years or older are reportedly less likely to have HγG [36], we found no significant difference was observed in the prevalence of HγG between younger patients (35.3%; *n* = 34; average age 49.7 ± 9.0 years) and older patients (30.3%; *n* = 33; average age 72.8 ± 6.3 years).

**Table 1:** Clinical characteristics of pSS patients (*n* = 67) involved in this study

Characteristic	
Female (%)	100
Age (mean ± SD years)	61.1 ± 14.0
Subjective ocular dryness (%)	100
Subjective oral dryness (%)	100
Presence of anti-SSA/Ro (%)	79.1
Presence of anti-SSB/La (%)	47.8
Presence of rheumatoid factor (%)	41.7
Serum IgG (mean ± SD; mg/dl)	1653 ± 587
IgG > 1700 mg/dl (%)	32.8
Serum IgA (mean ± SD; mg/dl)	289 ± 158
IgA > 410 mg/dl (%)	13.4
Serum IgM (mean ± SD; mg/dl)	107 ± 64.6
IgM > 220 mg/dl (%)	3.0
Steroid medication (%)	10.4
ESSDAI score	1.82 ± 2.42

Average serum levels of IgA and IgM in pSS patients were 289 ± 158 and 107 ± 64.6 mg/dl, respectively (Table 1). Serum IgM levels of sixty-five pSS patients (97%) were within the reference range of normal Japanese individuals (35–220 mg/dl, Table 1) [35], while serum IgA levels of 13.4% of 9 patients (13.4%) were above the reference range (110–410 mg/dl, Table 1) [35].

Seven patients among 67 patients received a low dose (7.2 mg/day) of prednisolone (PSL). Although the proportion of BR3-positive monocytes (BR3^+^/CD14^+^), serum IgG level, and ESSDAI score of these patients (49.9%, 2106 mg/dl and 3.86, respectively) were slightly higher than those of patients without the treatment (41.4%, 1600 mg/dl and 1.59), the differences were not statistically significant (*p* = 0.254, *p* = 0.083, and *p* = 0.092).

### Elevated expression of BAFF receptors, BR3 and TACI, in peripheral monocytes from pSS patients

BAFF and IL-6 are well known to play crucial roles in IgG production by B cells [7, 11, 13, 15, 29]. In addition, our previous studies indicate that peripheral monocytes produce sBAFF and IL-6 upon stimulation in vitro [28]. Moreover, we found that pSS monocytes show a robust increase in IL-6 production following stimulation with BAFF in vitro [28]. Therefore, we focused on monocytes in our exploration of the possible mechanisms underlying B cell activation.

**Fig. 1:**
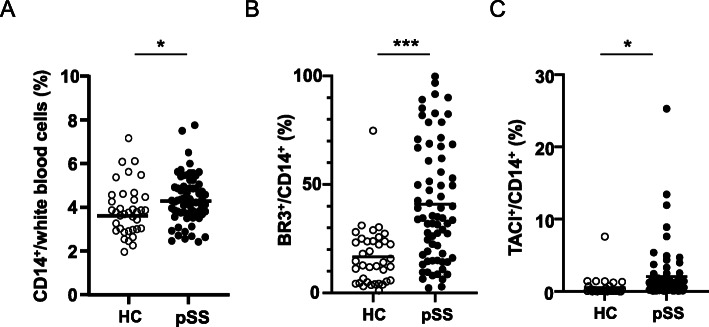
Proportion of CD14^+^ cells, BR3^+^/CD14^+^ cells, and TACI^+^/CD14^+^ cells in peripheral blood. Whole blood samples collected from HC (*n* = 37) and pSS patients (*n* = 67) were incubated with phycoerythrin-labeled anti-BR3 or phycoerythrin-labeled anti-TACI and Pacific blue-labeled anti-CD14 mAbs. Anti-CD14-positive cells (**a**), anti-CD14/anti-BR3 double-positive cells (**b**), and CD14/TACI double-positive cells (**c**) were analyzed by FACS. Horizontal lines indicate mean. *p < 0.05, ***p < 0.001

First, we measured the proportion of CD14^+^ cells among peripheral white blood cells. FACS analysis showed that the proportion of CD14^+^ cells in pSS patients (4.4 ± 1.1%) was slightly but significantly higher than that in HC (3.9 ± 1.2%; *p* = 0.017; Fig. 1a). These data suggest that the number of monocytes is abnormally elevated in pSS patients compared to HC.

We previously reported that IL-6 production by pSS monocytes was markedly enhanced following stimulation with sBAFF in vitro, which arises as a consequence of the elevated expression of BR3, a BAFF-specific receptor, in pSS monocytes [28]. Therefore, we quantitatively analyzed the proportion of BR3-positive cells among peripheral CD14^+^ cells (BR3^+^/CD14^+^) in whole blood from pSS patients and HC. The results clearly showed that the proportion of BR3-positive monocytes was significantly higher in pSS patients than in HC. Remarkably, the average BR^+^/CD14^+^ ratio in pSS patients was almost two-and-a-half-times (42.3 ± 26.9%) higher than that in HC at basal levels (16.6 ± 13.5%, *p* < 0.001; Fig. 1b). Then, we additionally compared the BR3^+^/CD14^+^ ratio among autoimmune diseases, such as systemic lupus erythematosus (SLE) and rheumatoid arthritis (RA). As shown in Supplementary Fig. 2, the BR3^+^/CD14^+^ ratio in pSS was significantly higher than those in SLE (untreated, 22.7 ± 21.8%, *p* = 0.0032) and RA (untreated, 19.9 ± 15.8%, *p* = 0.0018). In addition, no significant differences in the ratio were observed between SLE and HC (*p* = 0.099) or RA and HC (*p* = 0.235). These data suggest that the increased BR3^+^/CD14^+^ ratio is likely to be pSS specific. In addition, we analyzed the expression level of TACI (transmembrane activator and calcium-modulator and cyclophilin ligand (CAML) interactor), also known to bind to BAFF, on peripheral monocytes to verify the receptor specificity. As shown in Fig. 1c, the proportion of TACI-positive monocytes was also elevated in pSS monocytes as compared to HC (*p* = 0.014). The average TACI^+^/CD14^+^ ratio in pSS patients was 2.13 ± 3.98% which was markedly lower than that of BR3^+^/CD14^+^ ratio.

### Significance of BR3 in IL-6 production by pSS monocytes

**Fig. 2:**
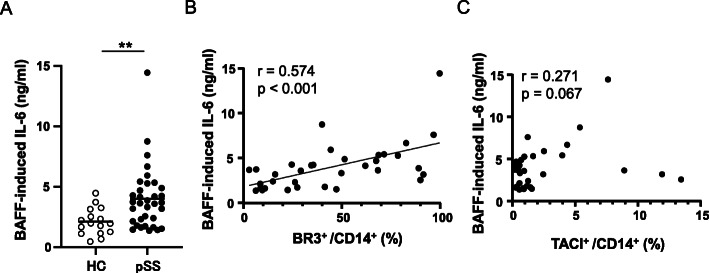
Correlation between the BR3^+^/CD14^+^ ratios or TACI^+^/CD14^+^ ratios and IL-6 production by BAFF-stimulated pSS monocytes. L-6 production by sBAFF-stimulated monocytes from HC (*n* = 16) and pSS patients (*n* = 34) was measured as described in the “Materials and methods” section (**a**). The peripheral BR3^+^/CD14^+^ ratios and TACI^+^/CD14^+^ ratios from pSS patients were calculated based on the FACS results. The concentration of IL-6 was plotted against BR3^+^/CD14^+^ (**b**) or TACI^+^/CD14^+^ ratio (**c**) for each patient. Pearson’s correlation analysis was used to examine the relationship between the parameters. A *p* value < 0.05 was considered significant

We previously reported that pSS monocytes showed a robust increase in IL-6 production upon stimulation with sBAFF in vitro [28]. We analyzed the potential relationship between the aberrations of pSS monocytes and overproduction of IL-6 by these cells in vitro. To this end, peripheral monocytes prepared from pSS patients and normal individuals were stimulated with sBAFF in vitro. The stimulation resulted in a striking increase of IL-6 production by monocytes and pSS monocytes showed a significantly higher production of IL-6 (4.01 ± 2.6 ng/ml) than normal monocytes (2.11 ± 1.11 ng/ml) upon stimulation (Fig. 2a; *p* = 0.0034). These results are consistent with our previous observations [28]. Notably, the amount of IL-6 produced by pSS monocytes upon stimulation with sBAFF was positively and significantly correlated with the BR3^+^/CD14^+^ ratio (*r* = 0.574, *p* < 0.001; Fig. 2b). In contrast, the proportion of TACI^+^/CD14^+^ ratio was not significantly correlated with the amount of IL-6 produced by BAFF-stimulated pSS monocytes (*r* = 0.271, *p* = 0.067; Fig. 2c). These findings indicate that IL-6 production was induced by BAFF-stimulated monocytes mainly via BR3. Moreover, the proportion of CD14^+^ cells among peripheral white blood cells from pSS patients did not correlate with the BR3^+^/CD14^+^ ratio and TACI^+^/CD14^+^ ratio (*r* = − 0.008, *p* = 0.473; *r* = 0.099, *p* = 0.217, respectively; Supplementary Fig. 3A and 3B). These data suggest that the robust production of IL-6 by pSS monocytes in vitro can mainly be ascribed to a larger population of BR3^+^ cells among CD14^+^ cells.

### Significance of BR3-positive monocytes in the clinical features of pSS patients

**Fig. 3:**
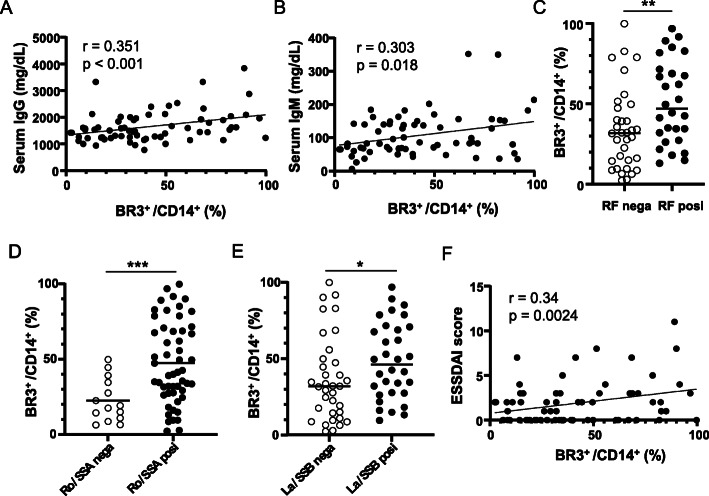
Correlation between the BR3^+^/CD14^+^ ratio and clinical features of patients with pSS. BR3^+^/CD14^+^ ratios in pSS patients were calculated based on the FACS results. Serum levels of IgG (**a**) and IgM (**b**) were plotted against the BR3^+^/CD14^+^ ratio for each patient. The ratios in patients with or without anti-Ro/SSA (**c**) and anti-La/SSB (**d**) antibodies are shown. The ratios were plotted against their ESSDAI score for each patient (**e**). Pearson’s correlation analysis was employed to evaluate the linear relationship between two continuous variables. Differences between the groups were statistically analyzed using the two-tailed Student’s *t* test for single comparison. *p < 0.05, ***p < 0.001

Following findings that the proportion of CD14^+^ cells and BR3^+^/CD14^+^ ratio was considerably elevated in pSS patients, we next examined whether these monocyte aberrations correlated with the clinical features of pSS that were involved in B cell activation. We found a positive and significant correlation between the BR3^+^/CD14^+^ ratio and serum levels of IgG (*r* = 0.351, *p* = 0.0018; Fig. 3a) and IgM (*r* = 0.303, *p* = 0.0064; Fig. 2b), but no correlation between the proportion of CD14^+^ cells and serum IgG (*r* = 0.05, *p* = 0.343; Supplementary Fig. 4A) and IgM (*r* = − 0.077, *p* = 0.269; Supplementary Fig. 4B) in pSS patients. These data strongly suggest that the expression of BR3 on pSS monocytes, rather than the number of monocytes, is related to the production of immunoglobulins. Next, we investigated the possible involvement of BR3^+^ monocytes in the production of autoantibodies, such as rheumatoid factor (RF), anti-Ro/SSA, and La/SSB antibodies. The BR3^+^/CD14^+^ ratio was significantly higher in serum RF-positive patients than in RF-negative patients (*p* = 0.0052; Fig. 3c). Similarly, the ratio was also significantly higher in serum anti-La/SSA-positive or anti-La/SSB-positive patients than in anti-La/SSA-negative (*p* = 0.0008; Fig. 3d) or anti-La/SSB-negative (*p* = 0.037; Fig. 3e) patients. Notably, the proportion of BR3^+^ monocytes was positively and significantly correlated with the patients’ ESSDAI score (*r* = 0.34, *p* = 0.0024; Fig. 3f). In contrast, TACI^+^/CD14^+^ ratio was not significantly correlated with serum levels of IgG and IgM (*r* = 0.14, *p* = 0.136; and *r* = 0.043, *p* = 0.369, respectively; Supplementary Fig. 5A and 5B) and ESSDAI score of patients (*r* = 0.134, *p* = 0.14; Supplementary Fig. 5C). Our findings suggest that elevated expression of BR3 in monocytes may contribute to not only B cell activation but also the clinical features observed in pSS patients.

### Stimulation of IgG production by B cells co-cultured with monocytes

**Fig. 4:**
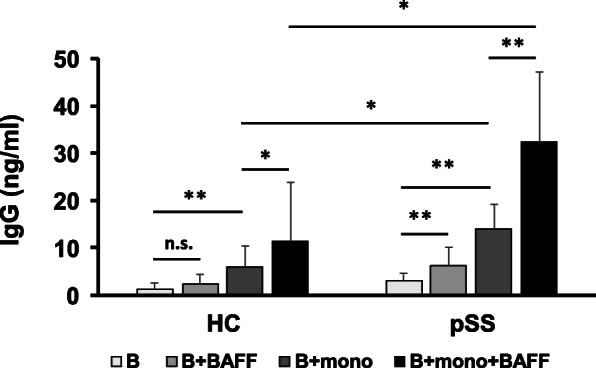
IgG production by B cells cultured in vitro. Monocytes and B cells were prepared from primary pSS patients (*n* = 10) and HC (*n* = 9). B cells (7.5 × 10^4^/ml) were cultured with or without monocytes (1.5 × 10^5^/ml) from the same subject in a 24-well plate using a transwell insert in the absence or presence of 2.0 μg/ml of sBAFF for 4 days. Concentrations of IgG in the culture supernatants were measured by ELISA. *p < 0.05, **p < 0.01

Together, the results described above suggest the possibility that BR3^+^ monocytes are involved in processes related to B cell activation, such as the overproduction of IgG by B cells. To examine this, we co-cultured monocytes and B cells in vitro using transwell insert and measured the amount of IgG in the culture supernatants by ELISA. Figure 4 shows that stimulation of pSS B cells with sBAFF slightly but significantly increased IgG production, while stimulation with sBAFF did not increase IgG production by B cells from HC (“B” vs. “B + sBAFF”: pSS *p* = 0.02, HC *p* = 0.153; Fig. 4). Furthermore, addition of autologous monocytes to the culture drastically enhanced IgG production by pSS B cells (“B” vs. “B + mono”, *p* = 0.004; Fig. 4). Notably, pSS B cells cultured with autologous monocytes produced significantly greater amounts of IgG than those from HC (“HC B + mono” vs. “pSS B + mono,” *p* = 0.019; Fig. 4). Supplementation of the co-culture with sBAFF further augmented IgG production by pSS B cells compared to HC (“pSS; B + mono + BAFF” vs “HC; B + mono + BAFF” *p* = 0.048; Fig. 4). The augmentation was statistically significant in both pSS patients (“B + mono” vs. “B + mono + sBAFF,” *p* = 0.005; Fig. 4) and HC (“B + mono” vs. “B + mono + sBAFF,” *p* = 0.024; Fig. 4). These data suggest that soluble factors produced by BAFF-stimulated monocytes may contribute to IgG production by B cells.

### Contribution of IL-6 derived from BAFF-stimulated monocytes to IgG production by B cells

**Fig. 5:**
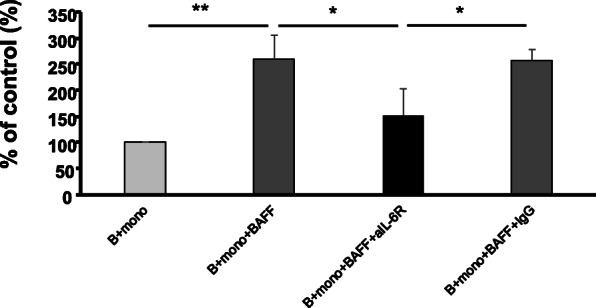
Suppressive effect of anti-IL-6 receptor antibody on IgG production by B cells co-cultured with monocytes. Monocytes and B cells from pSS patients (*n* = 4) were cultured as described in the legend of Fig. 4. Anti-IL-6 receptor antibody or isotype control antibody was added into the culture at day 0, and the culture supernatants were harvested on day 4. The amount of IgG produced by the cells in the supernatants was measured by ELISA, and the IgG level was normalized against levels in “B + mono”. *p < 0.05, **p < 0.01

To elucidate the mechanisms underlying the enhanced IgG production by pSS B cells when co-cultured with BAFF-stimulated monocytes, we added anti-human IL-6 receptor antibody to the culture system to normalize the IL-6 produced by monocytes. As shown in Fig. 5, the antibody partially but significantly inhibited IgG production (*p* = 0.028), while control IgG did not suppress the production.

Taken together, our findings suggest that soluble factors produced by BAFF-stimulated monocytes mediate IgG production by B cells in our co-culture system, and IL-6 is one of the candidates for B cell activation.

## Discussion

Accumulating evidence suggests that BAFF and IL-6 are involved in the development of pSS [17–22, 37, 38]. Given that these cytokines are produced by monocytes [11, 13, 39, 40], we speculated that monocytes are implicated in the pathogenesis of pSS and have reported several aberrations of pSS monocytes [28]. In the present study, we investigated the relationship between these aberrations and patients’ clinical features, in particular those related to B cell activation and subsequent antibody production which are deeply involved in the pathogenesis of pSS.

We found that the proportion of BR3^+^ monocytes was significantly higher in pSS patients than HC and was correlated with the patients’ serum titers of IgG and IgM and ESSDAI score. Moreover, BR3 expression in pSS monocytes was elevated in anti-Ro/SSA and/or anti-La/SSB antibody-positive patients compared to negative patients. The proportion of TACI^+^ monocytes was also elevated in pSS patients than HC; however, TACI^+^/CD14^+^ ratio was not significantly correlated with the patients’ serological data and clinical features. These data suggest that BAFF enhances IL-6 production in monocytes mainly through BR3. In our in vitro study, we found that the amount of IL-6 produced by BAFF-stimulated monocytes was correlated with the expression level of BR3 not with TACI level in pSS monocytes and that the amount of IgG produced by B cells was drastically enhanced when the cells were cultured with monocytes in the presence of BAFF. Given that IL-6 blockade using anti-IL-6 receptor antibody significantly inhibited IgG production by B cells that were co-cultured with BAFF-stimulated monocytes, we concluded that monocyte-derived IL-6 mediates IgG production by B cells in pSS.

We found that 32.8% of pSS patients enrolled in this study had HγG (Table 1). This incidence was comparable to that reported previously (39%) [36]. Unexpectedly, 13.4% of patients also had serum IgA levels above the reference range of normal Japanese individuals (Table 1) [35]. This incidence was higher than that reported previously (9%) [36], despite similar cut-off values for IgA in the present and previous study (410 mg/dl vs 463 mg/dL). Moreover, the serum level of IgG was positively and significantly correlated with serum IgA level in pSS patients (data not shown). No such correlation was observed between IgG and IgM and IgA and IgM (data not shown). Many lines of evidence suggest that IgG serum levels are significantly elevated in pSS patients as compared to HC [2] and that subsequently, serum IgA levels are increased in the patients [36]. Studies have reported that IgG-producing plasma cells accumulate in the germinal center formed in salivary glands of the patients [5], while IgA-producing plasma cells play an important role in the gastrointestinal mucosal defense system [41]. However, the mechanisms underlying the overproduction of IgG and IgA by B cell activation in pSS patients are not fully understood.

To elucidate the possible mechanism of B cell activation in pSS, we focused on the function of BAFF because BAFF plays a crucial role in B cell activation. BAFF is mainly produced by monocytes, macrophages, and dendritic cells and primarily binds its receptors on B cells to promote their proliferation and differentiation. Therefore, with the hypothesis that functionally impaired monocytes are involved in the symptoms of pSS, we measured the proportion of CD14^+^ cells, the main source of BAFF, among peripheral white blood cells. FACS analysis revealed that the proportion of CD14^+^ cells in pSS patients was significantly higher than that of HC (Fig. 1a). Despite the significant increase, however, we found no correlation between the proportion of CD14^+^ cells and serum levels of IgG (*r* = 0.05, *p* = 0.343; Supplementary Fig. 4A) or IgM (*r* = − 0.077, *p* = 0.269; Supplementary Fig. 4B). BAFF binds its receptors, such as BR3, TACI, and BCMA. Among them, BR3, a specific receptor for BAFF, has been reported to be expressed on B cells, activated T cells, and dendritic cells and that plays an important role in B cell activation by binding with BAFF [7]. It has been reported that BR3 expression in B cells is downregulated when B cells differentiate to plasma cells, whereas TACI expression is upregulated instead [42]. These reports suggest that BR3 and TACI strongly contribute to B cell activation. Given that BR3 and TACI are expressed on pSS monocytes [28], we subsequently examined the proportion of BR3-positive cells (BR3^+^/CD14^+^) and TACI-positive cells (TACI^+^/CD14^+^) among peripheral CD14^+^ cells. Interestingly, both ratios of BR3^+^/CD14^+^ and TACI^+^/CD14^+^ were significantly elevated in pSS patients compared to HC (Fig. 1b, c). Remarkably, the BR3^+^/CD14^+^ ratio was positively correlated with serum levels of IgG and IgM in pSS patients (Fig. 3a, b). In contrast, there was no correlation between the TACI^+^/CD14^+^ ratio and serum levels of IgG and IgM of the patients (Supplementary Fig. 5A and 5B). These data raise the possibility that elevated expression of BR3 is a noteworthy aberration of pSS monocytes.

Notably, we found that the BR3^+^/CD14^+^ ratio was significantly higher in RF-positive patients as compared to RF-negative patients (Fig. 3c). In addition, the ratio was also higher than in serum anti-Ro/SSA antibody-positive patients than negative patients (Fig. 3d) and that serum anti-La/SSB-positive patients possessed a significantly greater proportion of BR3^+^ monocytes than anti-La/SSB-negative patients (Fig. 3e). Moreover, the BR3^+^/CD14^+^ ratio was positively and significantly correlated with the patients’ ESSDAI score (Fig. 3f). Our data suggest that elevated BR3 expression in monocytes may contribute to B cell activation and disease activity.

One may claim that the abnormalities of pSS patients are mere consequences of the difference in average age between pSS patients (61.1 years) and HC (41.6 years) enrolled in this study. However, serum IgG levels (Table 1) and the proportion of CD14^+^ cells (Fig. 1a) were age-independent (*r* = 0.026, *p* > 0.10 for IgG; *r* = 0.04, *p* > 0.10 for CD14^+^; data not shown). Further, surprisingly and unexpectedly, the BR3^+^/CD14^+^ ratio was negatively and significantly correlated with age among pSS patients (Supplementary Fig. 6). These data strongly suggest that the increased BR3^+^/CD14^+^ ratio in pSS patients (Fig. 1b) was not due to the higher average age of pSS patients. Therefore, it is unlikely that age is a crucial factor for these abnormalities.

These results prompted us to investigate the cellular mechanisms underlying the correlation between BR3^+^ monocytes and B cell function. First, we examined the responses of monocytes to stimulation with sBAFF in vitro and the correlation between the proportion of BR3^+^ monocytes and the amount of IL-6 produced by cells stimulated with BAFF. In our previous study, we found that pSS monocytes showed a robust increase in IL-6 production [28]. In the present study, the amount of IL-6 produced in vitro by sBAFF-stimulated pSS monocytes was positively and significantly correlated with the BR3^+^/CD14^+^ ratio (*r* = 0.574, *p* = 0.0004; Fig. 2b). These data, in conjunction with the data presented in Fig. 3a (BR3^+^/CD14^+^ ratio vs serum IgG; *r* = 0.351, *p* = 0.0036), collectively suggest that pSS monocytes are involved in the overproduction of IgG by B cells.

Based on these findings, we hypothesized that the activation of B cells by monocytes requires close interaction between these cells. To examine this hypothesis, we co-cultured pSS B cells and autologous monocytes with or without BAFF using a transwell insert in vitro. Monocytes significantly enhanced IgG production by B cells (Fig. 4). Addition of sBAFF to the co-culture further augmented IgG production. These data suggest that sBAFF activates B cells through factors secreted by monocytes, such as IL-6, and contributes to the overproduction of IgG by B cells. As shown in Fig. 5, addition of an anti-IL-6R antibody to the co-culture significantly inhibited IgG production, while control IgG did not suppress that production. Taken together, our data suggest that IL-6 produced by BAFF-stimulated BR3^+^ monocytes is one of the candidate factors which accelerates IgG production by B cells.

Some limitations of this study warrant mention. First, since the disease activity of the patients enrolled in this study was relatively moderate (average ESSDAI score, 1.82 ± 2.42), further investigation of patients with higher disease activity may be required. Second, although our cross-sectional research indicates that the elevated expression of BR3 in monocytes correlates with the clinical features of pSS, additional longitudinal studies and elucidation of regulatory mechanisms of BR3 expression in monocytes will be important for understanding the contribution of BR3-expressing monocytes to changes in the disease. Third, to clarify the involvement of BAFF-BR3 axis in lesion of tissues, such as salivary glands and lachrymal glands, further studies using tissue samples from patients may be required.

## Conclusion

The results of our study collectively suggest that aberrations of monocytes may underlie the activation of B cells and the clinical features observed in pSS patients. In addition, we found that IL-6 derived from BAFF-stimulated monocytes is involved in the overproduction of IgG by B cells. Our findings raise the possibility that inhibition of BR3, which will suppress functions of not only B cells but also monocytes, is one of the promising therapies for the treatment of pSS.

## Supplementary information

**Additional file 1: Figure S1.** Definition of BR3^+^ cells among CD14^+^ monocytes by FACS analysis. The proportion of BR3^+^ cells among CD14^+^ monocytes was analyzed by FACS as described in Materials and Methods. Representative data of BR3^+^CD14^+^ monocytes in HC (A) and pSS patient (B) are shown. (PPTX 95 kb)

**Additional file 2: Figure S2.** Comparison of the BR3^+^/CD14^+^ ratio in peripheral blood among HC and patients with pSS, SLE and RA. Whole blood samples collected from HC (*n* = 37), pSS patients (*n* = 67), SLE patients (untreated, *n* = 20) and RA patients (untreated, *n* = 14) were incubated with phycoerythrin-labeled anti-BR3 and Pacific Blue-labeled anti-CD14 mAbs. The BR3^+^/CD14^+^ ratio was analyzed by FACS. Horizontal lines indicate mean. ** *p*<0.01, *** *p*<0.001. (PPTX 49 kb)

**Additional file 3: Figure S3.** Correlation between the proportion of CD14^+^ monocytes and BR3^+^/CD14^+^ and TACI^+^/CD14^+^ in patients with pSS. The proportion of CD14^+^ monocytes among peripheral white blood cells, BR3^+^/CD14^+^ ratios and TACI^+^/CD14^+^ ratios of pSS patients were calculated based on the results of FACS. BR3^+^/CD14^+^ ratios (A) and TACI^+^/CD14^+^ ratios (B) were plotted against the proportion of CD14^+^ monocytes for each patient. Pearson’s correlation analysis was used to examine the relationship between the parameters. A *p* value < 0.05 was considered significant. (PPTX 58 kb)

**Additional file 4: Figure S4.** Correlation between the proportion of CD14^+^ monocytes and serum IgG and IgM levels in patients. The proportion of CD14^+^ monocytes among peripheral white blood cells of pSS patients was calculated based on the results of FACS. Serum levels of IgG (A) and IgM (B) were plotted against the proportion of CD14^+^ monocytes for each patient. Pearson’s correlation analysis was used to examine the relationship between the parameters. A p value < 0.05 was considered significant. (PPTX 56 kb)

**Additional file 5: Figure S5.** Correlation between the TACI^+^/CD14^+^ ratios and clinical features of patients with pSS. TACI^+^/CD14^+^ ratios in pSS patients were calculated based on the FACS results. Serum levels of IgG (A) and IgM (B) were plotted against the TACI^+^/CD14^+^ ratios for each patient. The ratios were plotted against their ESSDAI score for each patient (C). Pearson’s correlation analysis was employed to evaluate the linear relationship between two continuous variables. (PPTX 67 kb)

**Additional file 6: Figure S6.** Correlation between the BR3^+^/CD14^+^ ratios and the age of patients with pSS. The BR3^+^/CD14^+^ ratios of pSS patients were calculated based on the FACS results. The BR3^+^/CD14^+^ ratio was plotted against the age for each patient. Pearson’s correlation analysis was examined for statistical significance between the groups. A p value < 0.05 was considered significant. (PPTX 49 kb)

## Data Availability

All data generated and analyzed in this study are disclosed in this article.

## Abbreviations

pSS: Primary Sjögren’s syndrome
BAFF: B cell activating factor
sBAFF: Soluble BAFF
HγG: Hypergammaglobulinemia
BR3: BAFF receptor
TACI: Transmembrane activator and calcium-modulator and cyclophilin ligand (CAML) interactor
BCMA: B cell maturation antigen
