# Supplementary figures and images for: Elevated expression of BAFF receptor, BR3, on monocytes correlates with B cell activation and clinical features of patients with primary Sjögren’s syndrome
Source: Arthritis Res Ther. 2020 Jun 23;22:157. doi: 10.1186/s13075-020-02249-1 (PMC7310340; doi:10.1186/s13075-020-02249-1)

## Slide 1
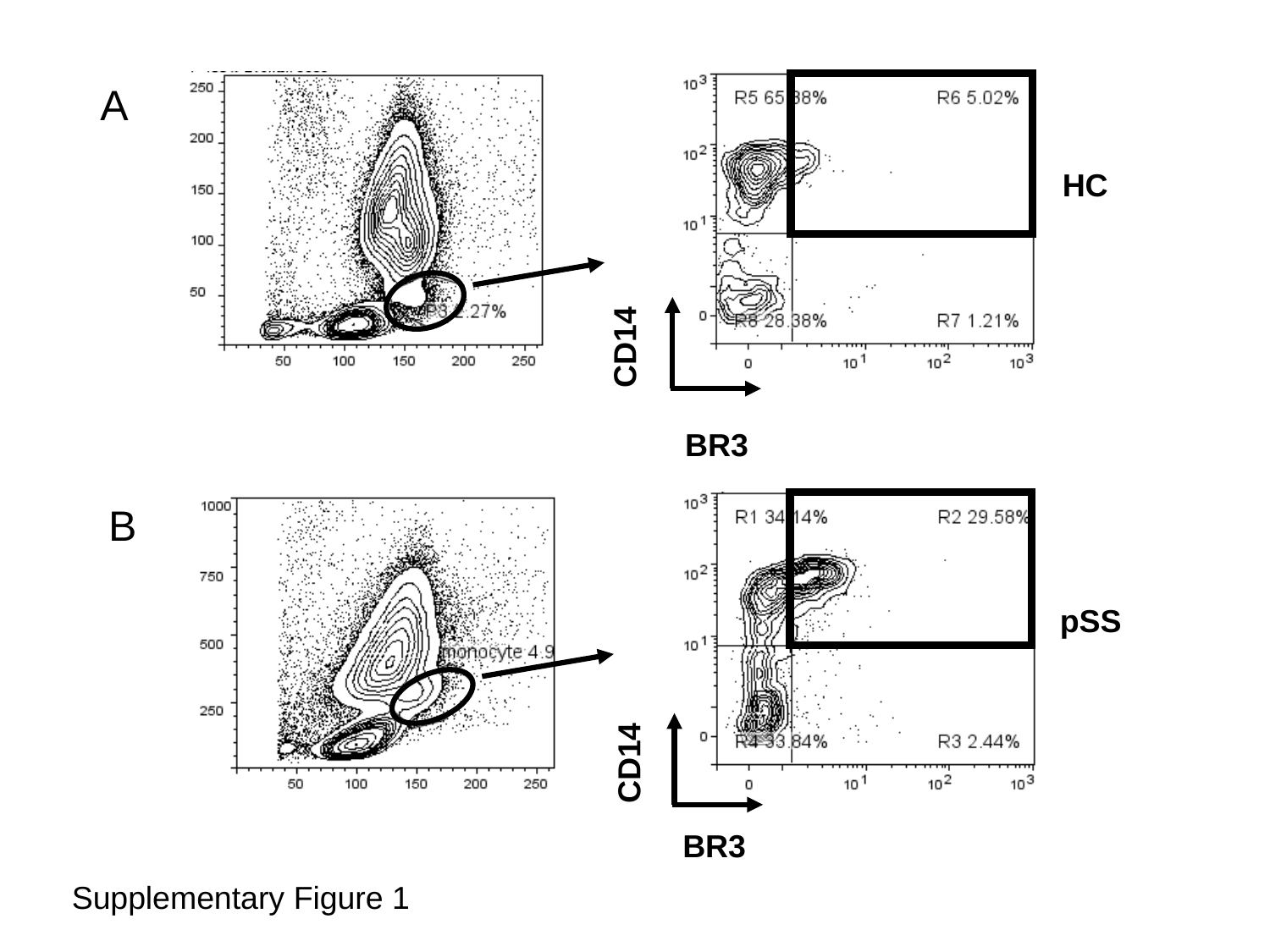

A
CD14
BR3
HC
B
CD14
BR3
pSS
Supplementary Figure 1

Supplement: Supplementary file 1 — Additional file 1: Figure S1. Definition of BR3+ cells among CD14+ monocytes by FACS analysis. The proportion of BR3+ cells among CD14+ monocytes was analyzed by FACS as described in Materials and Methods. Representative data of BR3+CD14+ monocytes in HC (A) and pSS patient (B) are shown. (PPTX 95 kb) [file 13075_2020_2249_MOESM1_ESM.pptx]

## Slide 1
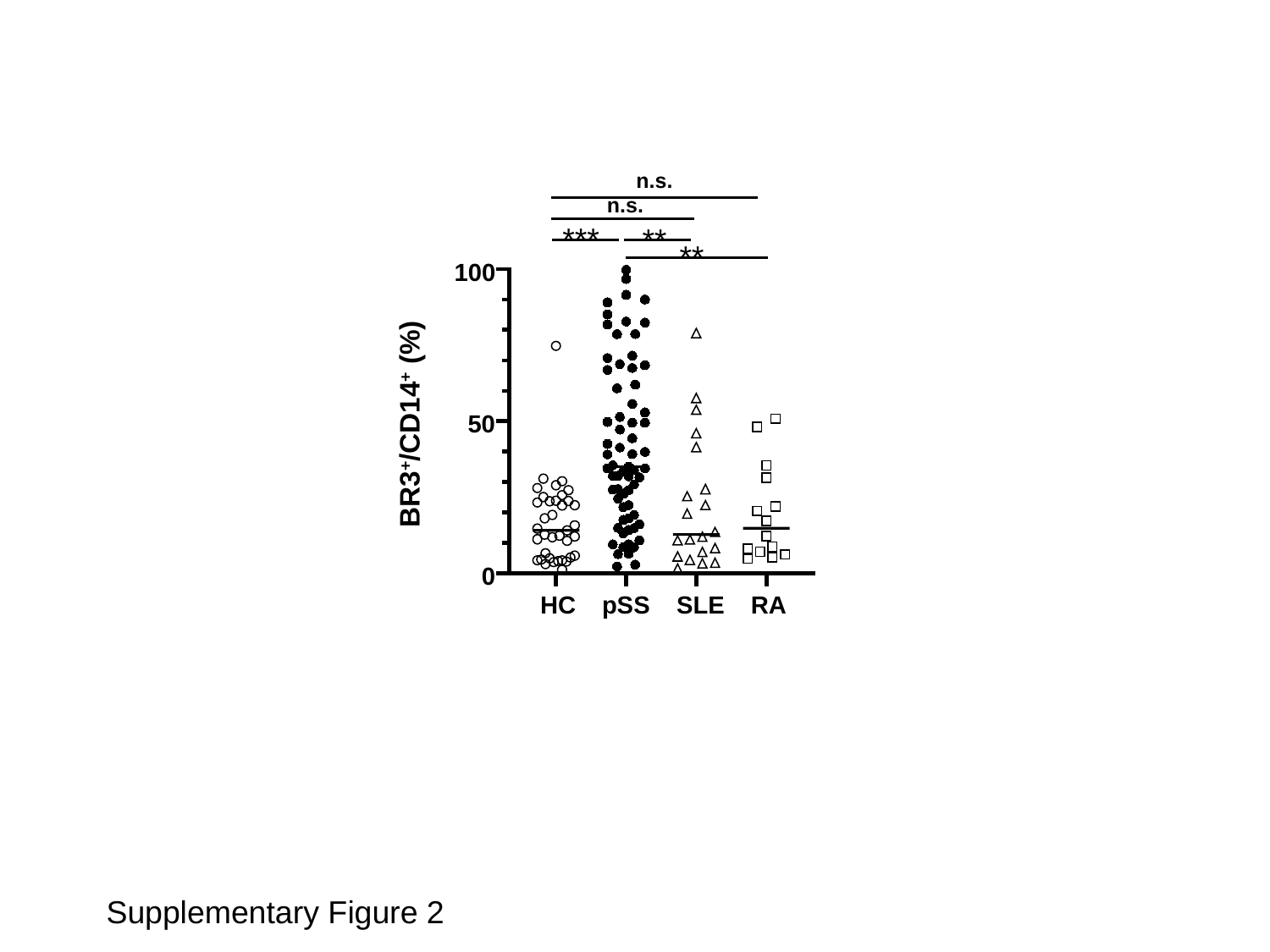

n.s.
n.s.
***
**
**
100
BR3+/CD14+ (%)
50
0
HC
pSS
SLE
RA
Supplementary Figure 2

Supplement: Supplementary file 2 — Additional file 2: Figure S2. Comparison of the BR3+/CD14+ ratio in peripheral blood among HC and patients with pSS, SLE and RA. Whole blood samples collected from HC (n = 37), pSS patients (n = 67), SLE patients (untreated, n = 20) and RA patients (untreated, n = 14) were incubated with phycoerythrin-labeled anti-BR3 and Pacific Blue-labeled anti-CD14 mAbs. The BR3+/CD14+ ratio was analyzed by FACS. Horizontal lines indicate mean. ** p<0.01, *** p<0.001. (PPTX 49 kb) [file 13075_2020_2249_MOESM2_ESM.pptx]

## Slide 1
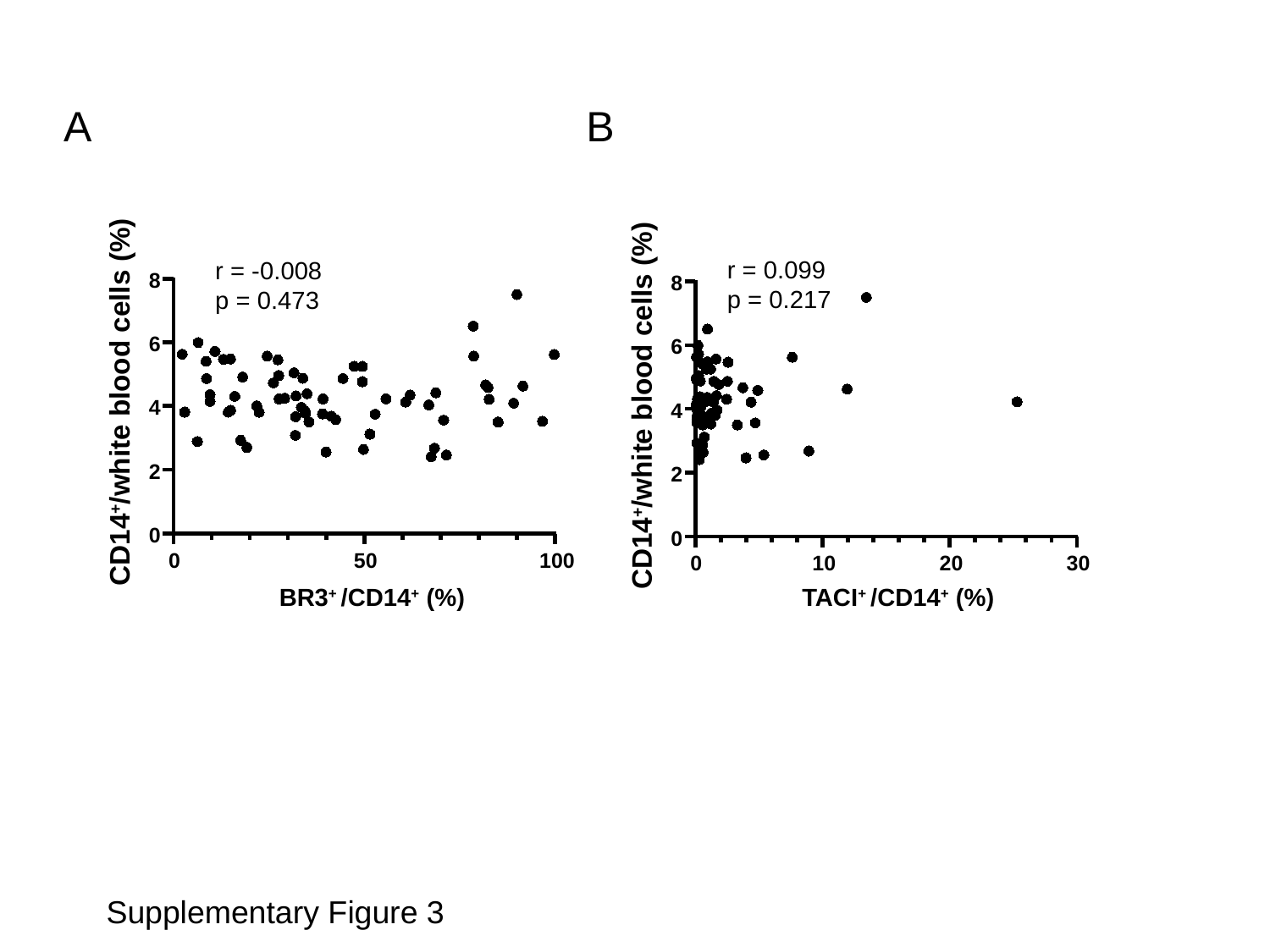

A
B
r = -0.008
p = 0.473
8
6
CD14+/white blood cells (%)
4
2
0
0
50
100
BR3+ /CD14+ (%)
r = 0.099
p = 0.217
8
6
4
2
0
0
10
20
30
TACI+ /CD14+ (%)
CD14+/white blood cells (%)
Supplementary Figure 3

Supplement: Supplementary file 3 — Additional file 3: Figure S3. Correlation between the proportion of CD14+ monocytes and BR3+/CD14+ and TACI+/CD14+ in patients with pSS. The proportion of CD14+ monocytes among peripheral white blood cells, BR3+/CD14+ ratios and TACI+/CD14+ ratios of pSS patients were calculated based on the results of FACS. BR3+/CD14+ ratios (A) and TACI+/CD14+ ratios (B) were plotted against the proportion of CD14+ monocytes for each patient. Pearson’s correlation analysis was used to examine the relationship between the parameters. A p value < 0.05 was considered significant. (PPTX 58 kb) [file 13075_2020_2249_MOESM3_ESM.pptx]

## Slide 1
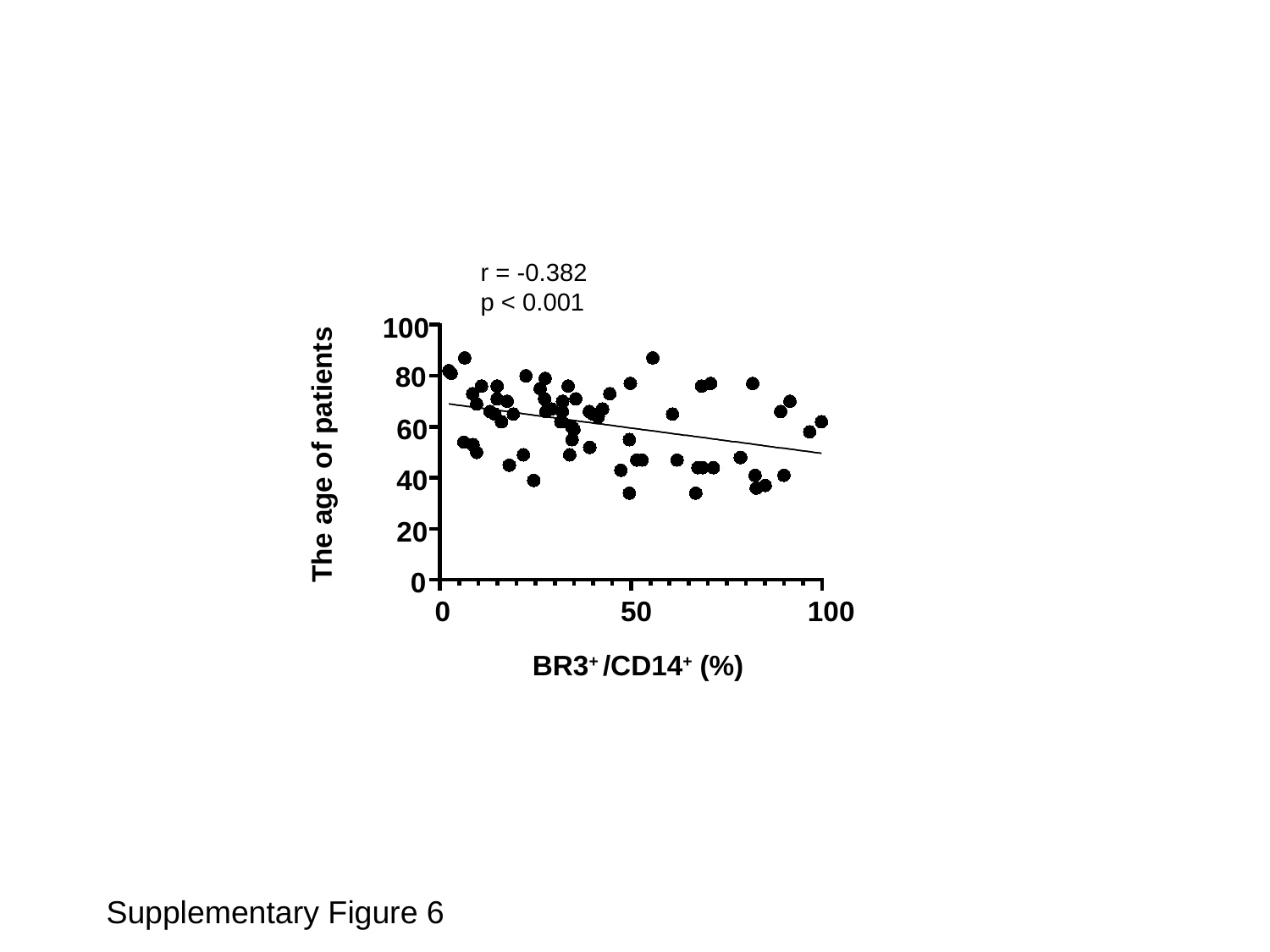

r = -0.382
p < 0.001
100
80
60
The age of patients
40
20
0
0
50
100
BR3+ /CD14+ (%)
Supplementary Figure 6

Supplement: Supplementary file 6 — Additional file 6: Figure S6. Correlation between the BR3+/CD14+ ratios and the age of patients with pSS. The BR3+/CD14+ ratios of pSS patients were calculated based on the FACS results. The BR3+/CD14+ ratio was plotted against the age for each patient. Pearson’s correlation analysis was examined for statistical significance between the groups. A p value < 0.05 was considered significant. (PPTX 49 kb) [file 13075_2020_2249_MOESM6_ESM.pptx]
