# Supplementary material for: Elevated expression of BAFF receptor, BR3, on monocytes correlates with B cell activation and clinical features of patients with primary Sjögren’s syndrome
Source: Arthritis Res Ther. 2020 Jun 23;22:157. doi: 10.1186/s13075-020-02249-1 (PMC7310340; doi:10.1186/s13075-020-02249-1)
Supplement: Supplementary file 4 — Additional file 4: Figure S4. Correlation between the proportion of CD14+ monocytes and serum IgG and IgM levels in patients. The proportion of CD14+ monocytes among peripheral white blood cells of pSS patients was calculated based on the results of FACS. Serum levels of IgG (A) and IgM (B) were plotted against the proportion of CD14+ monocytes for each patient. Pearson’s correlation analysis was used to examine the relationship between the parameters. A p value < 0.05 was considered significant. (PPTX 56 kb) [file 13075_2020_2249_MOESM4_ESM.pptx]

## Slide 1
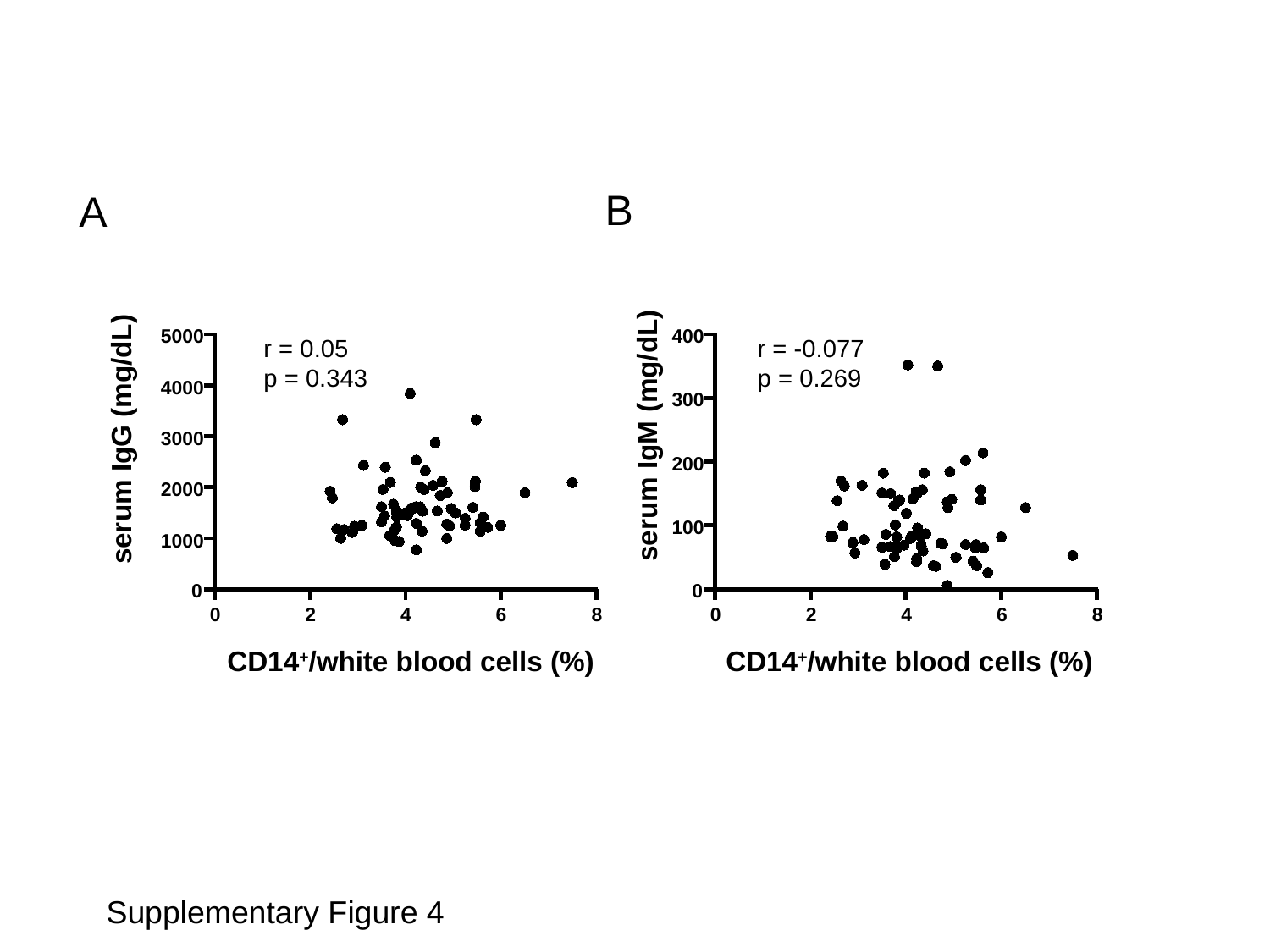

B
A
5000
400
r = 0.05
p = 0.343
r = -0.077
p = 0.269
4000
300
serum IgM (mg/dL)
serum IgG (mg/dL)
3000
200
2000
100
1000
0
0
0
2
4
6
8
0
2
4
6
8
CD14+/white blood cells (%)
CD14+/white blood cells (%)
Supplementary Figure 4
