# Supplementary material for: Elevated expression of BAFF receptor, BR3, on monocytes correlates with B cell activation and clinical features of patients with primary Sjögren’s syndrome
Source: Arthritis Res Ther. 2020 Jun 23;22:157. doi: 10.1186/s13075-020-02249-1 (PMC7310340; doi:10.1186/s13075-020-02249-1)
Supplement: Supplementary file 5 — Additional file 5: Figure S5. Correlation between the TACI+/CD14+ ratios and clinical features of patients with pSS. TACI+/CD14+ ratios in pSS patients were calculated based on the FACS results. Serum levels of IgG (A) and IgM (B) were plotted against the TACI+/CD14+ ratios for each patient. The ratios were plotted against their ESSDAI score for each patient (C). Pearson’s correlation analysis was employed to evaluate the linear relationship between two continuous variables. (PPTX 67 kb) [file 13075_2020_2249_MOESM5_ESM.pptx]

## Slide 1
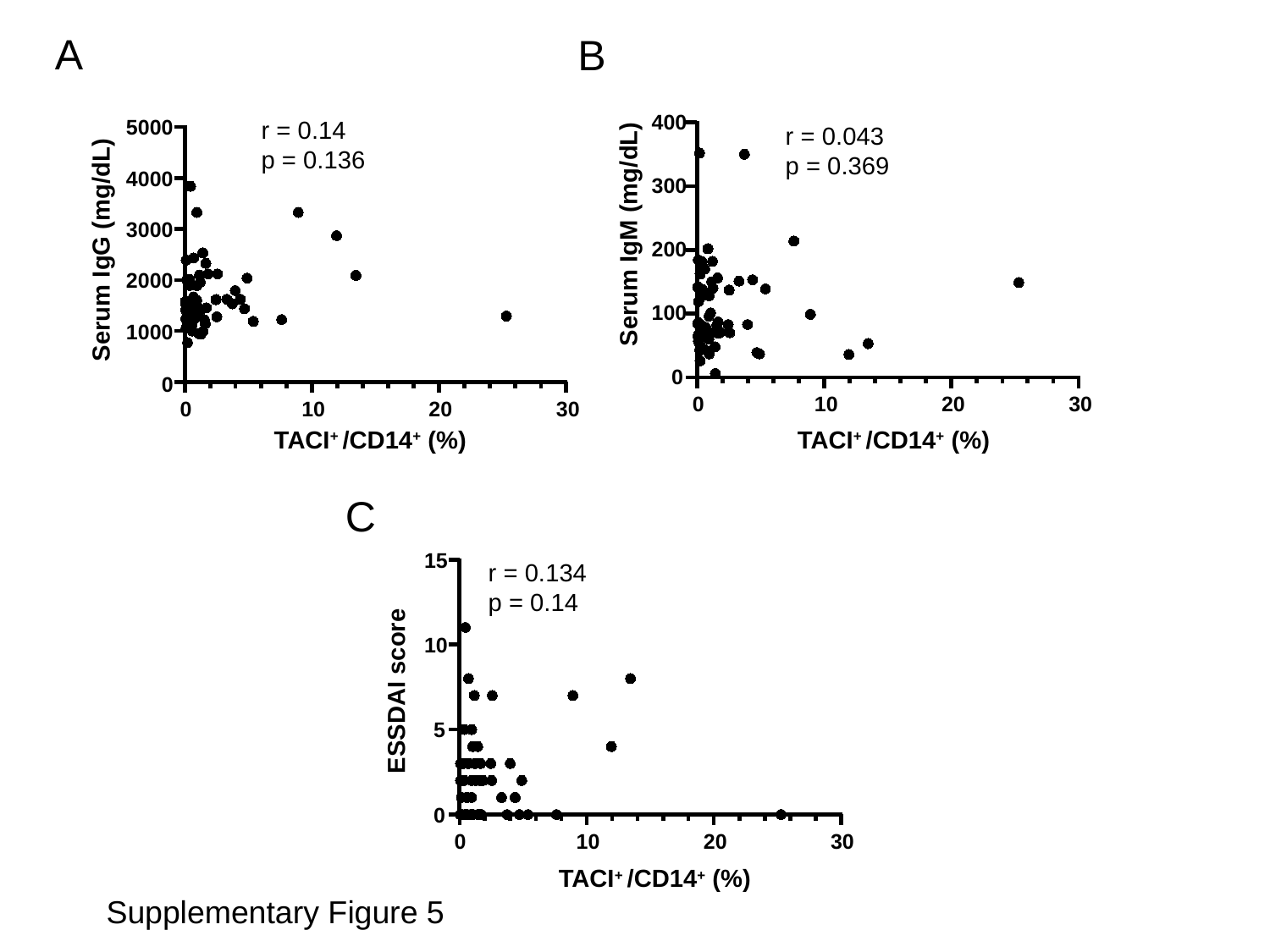

A
B
r = 0.14
p = 0.136
5000
4000
3000
2000
1000
0
0
10
20
30
TACI+ /CD14+ (%)
400
300
200
100
0
0
10
20
30
r = 0.043
p = 0.369
TACI+ /CD14+ (%)
Serum IgM (mg/dL)
Serum IgG (mg/dL)
C
15
r = 0.134
p = 0.14
10
ESSDAI score
5
0
0
10
20
30
TACI+ /CD14+ (%)
Supplementary Figure 5
